# Caregiver perceptions of child development in rural Madagascar: a cross-sectional study

**DOI:** 10.1186/s12889-019-7578-3

**Published:** 2019-09-11

**Authors:** Esther O. Chung, Lia C. H. Fernald, Emanuela Galasso, Lisy Ratsifandrihamanana, Ann M. Weber

**Affiliations:** 1 Department of Epidemiology, Gillings School of Global Public Health, University of North Carolina-Chapel Hill, McGavran-Greenberg Hall, CB# 7435, Chapel Hill, NC 27599-7435 USA; 2 Division of Community Health Sciences, School of Public Health, University of California, Berkeley, USA; 3 Development Research Group, The World Bank, Washington DC, USA; 4 Higher Institute of Social Work, Catholic University of Madagascar, Antananarivo, Madagascar; 5 School of Community Health Sciences, University of Nevada, Reno, Reno, USA

**Keywords:** Caregiver perceptions, Intelligence, Ages and stages questionnaire: Inventory, Early child development, Madagascar, Sub-Saharan Africa

## Abstract

**Background:**

Human capital (the knowledge, skills, and health that accumulate over life) can be optimized by investments in early childhood to promote cognitive and language development. Parents and caregivers play a crucial role in the promotion and support of cognitive development in their children. Thus, understanding caregiver perceptions of a child’s capabilities and attributes, including intelligence, may enhance investments early in life. To explore this question, we asked caregivers to rank their child’s intelligence in comparison with other children in the community, and compared this ranking with children’s scores on an assessment of developmental abilities across multiple domains.

**Methods:**

Our study examined cross-sectional data of 3361 children aged 16–42 months in rural Madagascar. Child intelligence, as perceived by their caregiver, was captured using a ladder ranking scale based on the MacArthur Scale for Subjective Social Status. Children’s developmental abilities were assessed using scores from the Ages and Stages Questionnaire: Inventory (ASQ-I), which measures cognitive, language, and socio-emotional development. Ranked percentiles of the ASQ-I were generated within communities and across the whole sample. We created categories of under-estimation, matched, and over-estimation by taking the differences in rankings between caregiver-perceived child intelligence and ASQ-I. Child nutritional status, caregiver belief of their influence on child intelligence, and sociodemographic factors were examined as potential correlates of discordance between the measures using multinomial logistic regressions.

**Results:**

We found caregiver perceptions of intelligence in Madagascar did not align consistently with the ASQ-I, with approximately 8% of caregivers under-estimating and almost 50% over-estimating their children’s developmental abilities. Child nutritional status, caregiver belief of their influence on child intelligence, caregiver education, and wealth were associated with under- or over-estimation of children’s developmental abilities.

**Conclusions:**

Our findings suggest parents may not always have an accurate perception of their child’s intelligence or abilities compared with other children. The results are consistent with the limited literature on parental perceptions of child nutrition, which documents a discordance between caregiver perceptions and objective measures. Further research is needed to understand the common cues caregivers that use to identify child development milestones and how these may differ from researcher-observed measures in low-income settings.

**Trial registration:**

Current Controlled Trials ISRCTN14393738. Registered June 23, 2015.

**Electronic supplementary material:**

The online version of this article (10.1186/s12889-019-7578-3) contains supplementary material, which is available to authorized users.

## Background

Across the world, over 250 million children under the age of 5 years are at risk of not achieving their full developmental potential because they live in poverty and/or are stunted (low height-for-age) [1]. The first few years of life are a critical time period in which rapid growth and development occur in the brain, and important neural networks and domains reach peak development [2]. Early child development (ECD) is typically characterized by activity in four areas: cognition, motor, language, and socioemotional domains. Exposure to risk factors, such as poverty, during this time period can have damaging physiological effects on the brain, which can affect later adult health and contribute to economic repercussions such as loss of productivity and workforce participation [3–5].

Human capital investments (i.e. in knowledge, skills, and health) early in life are important for future educational attainment, workforce participation, and productivity. Parental investment patterns are likely driven by beliefs and perceptions of children’s development trajectories, which depend directly on the information and signals that parents receive about their child and the comparison of their child’s abilities relative to other children. This feedback could take many forms, and could come from direct observation by the parents or explicit feedback from a child’s teacher, tutor, or other caregiver.

Parental perceptions of developmental progress in early childhood have been associated with parenting practices and later child development outcomes. In high-income countries, mothers who have greater knowledge of child development interact with their children more positively [6, 7] and are more likely to provide cognitive stimulation to their children [8]. For example, among preterm infants in the United States, maternal knowledge of developmental norms and milestones was associated with a higher-quality home environment, reduced child behavior problems, and improved cognitive development [9]. Prior research in Israel and Spain has also demonstrated that more positive maternal perceptions of their infants in relation to an average baby were associated with improved psychomotor and cognitive development of the baby in the first year of life [10, 11]. In other studies from the U.S. and Germany, accurate parental perceptions of early child developmental trajectories also predicted more positive temperament and behavior during school-aged years [12–14]. In another study from the U.S., parental beliefs about how well their child generally learns, thinks, and solves problems in relation to other similarly aged children was strongly associated with the child’s math and reading test scores relative to children in the same school on a school-administered test [15], and parents who believed their child was above average relative to other children the same age invested less (e.g. tutoring, help with homework) than other parents.

While research from lower-income countries is limited, a handful of studies suggests that parental perceptions are just as important for child development in the context of poverty. For example, Nigerian children aged 22–26 months who were rated by their parents as more responsible (i.e. able to purchase items or retrieve specific objects) had greater cognitive scores than children who were rated as less responsible [16]. Research from Malawi documented that inaccurate parental perceptions about children’s academic ability in school, especially among the poor, have important consequences on their educational decisions for their children [17]. Namely, when parents were provided with clear and understandable school performance information, they reallocated resources and school enrollment for higher-performing children.

Taken together, the existing research highlights how parents utilize indicators of their child’s abilities to inform educational investments. Therefore, understanding parental perceptions of children’s developmental abilities, including intelligence, is important for informing effective parenting programs; however, research in low-income contexts is limited. Existing studies from LMICs primarily focus on perceptions of weight and nutritional status [18–23], and in general, find parental perceptions in low-resource settings do not align with objective measures. These misperceptions may lead to behaviors and practices that have long-term negative consequences for child health and development.

The present study is based in Madagascar, a low-income country where 77.8% of the population lives on less than $1.90 a day [24] and where 49.2% of children under 5 years are stunted [25]. The government’s nutritional program has been in effect since 1999 and is founded on frontline community health workers hosting monthly growth monitoring sessions, during which they also provide mothers of children under 5 years with nutrition, feeding, and hygiene education. To examine the difference between Malagasy caregivers’ perceptions of child intelligence and developmental abilities, we asked caregivers to rank their child’s intelligence in comparison with other children in the community, and compared this ranking with how children scored on the Ages and Stages Questionnaire: Inventory (ASQ-I), an assessment of children’s developmental abilities across multiple domains including gross and fine motor, language, problem-solving, and social skills. In Malagasy, the term ‘intelligence’ translates to ‘sharp mind’ and caregivers generally understand it as a broad concept of child development that includes motor skills, problem solving, socio-emotional skills, language and cognitive capacity. Given that the Malagasy use of the term ‘intelligence’ encompasses similar constructs as the ASQ-I, our objectives were to 1) compare caregiver-perceived child intelligence in relation to other children in the community with how children scored on the ASQ-I, and 2) explore what child, caregiver, and household factors were associated with discordance between the two measures.

## Methods

### Study setting and design

Given that specific developmental domains of cognition, language, vision, and hearing largely occur in the early years of life [26], we focus on children under 4 years of age. Cross-sectional data of rural women and children aged 16–42 months were collected as part of the endline evaluation of a cluster-randomized controlled trial in Madagascar in 2016. The sample represents five regions of the country, which were selected because they have some of the highest rates of stunting and food insecurity in Madagascar: Amoron’ i Mania, Androy, Atsimo Atsinanana, Haute Matsiatra, and Vatovavy-Fitovinany. A more detailed description of the study design and sampling is available elsewhere [27]. Briefly, the five-arm trial tested the effects of intensive nutrition counseling, lipid-based supplementation, or a home visit parenting program on child growth and development. All pregnant women and women with age-eligible children for the intervention were eligible to participate in the trial. A total of 3560 caregivers and children were interviewed and assessed. The measure of ECD, the ASQ-I, was complete for 3533 children. Missing values for caregiver age (*N* = 82), caregiver education (*N* = 14), and child birth order (*N* = 1) were imputed using baseline and midline values. After imputation, complete data on child-, caregiver-, and household-level characteristics were available for 3361 children.

### Outcome

#### Caregiver-perceived intelligence

We modeled our caregiver-perceived intelligence scale on the existing MacArthur Scale for Subjective Social Status used in adults and adolescents and measures an individual’s perceived economic status [28]. The MacArthur Scale has been shown to be an independent predictor of adult health [29], adolescent self-rated health [30], and adolescent risky behaviors [31]. In the present study, caregivers were asked to look at a picture of a ladder with rungs labeled from 1 to 7, and asked: “At the top of the ladder are the children with the best intelligence status and at the bottom are children with the worst intelligence status. In your opinion, where is your child on this scale?” Caregivers were asked to focus specifically on the target child of the study and rank the child in relation to other children in the community using their own definition of intelligence. Prior to the survey administration, this scale was extensively pre-tested and the terms were discussed by investigators. The terms “Faharanitantsaina” (intelligence, or having a ‘sharp mind’) and “Maranitsaina” (to be intelligent) were selected and are commonly used terms in official Malagasy. In focus groups during the pre-testing, the term ‘intelligence’ was clearly perceived and understood by main caregivers as a broad concept of intelligence and child development that encompasses motor skills, problem solving, socio-emotional skills, language and cognitive capacity. In the instructions, interviewers were careful in asking caregivers to use their own definition of intelligence. Therefore, the subjective ranking was a function of individual and community characteristics.

#### Developmental abilities

Child developmental abilities were assessed using the ASQ-I, an instrument focused on developmental milestones for children aged 1–54 months across five domains: communication, gross motor, fine motor, problem solving, and personal-social. At the time of this study, the authors were given access to the unpublished ASQ-I instrument courtesy of the Ages and Stages Questionnaire (ASQ) group at the University of Oregon. The validity and reliability of the ASQ-I has since been published [32], and has also been adapted and validated in China [33]. To adapt the ASQ-I to Madagascar, the authors worked in an iterative process for any changes to item ordering, text adaptations, administration protocols for Madagascar, and coding of items. One of the study authors [LR], a local Malagasy psychologist translated and adapted it to the local context, and incorporated interviewer-observed items. The ASQ-I was then back-translated and reviewed by the ASQ group in Oregon. Three iterations of piloting, testing, and updating occurred before the instruments were finalized. For each ASQ-I domain, a summary score was created, and a total ASQ-I score was calculated by summing across the domain-specific scores. The total, continuous score was age-standardized and controlled for interviewer. In order to compare this scale to the caregiver-perceived intelligence measure, we created seven percentile-based, rank-ordered (from 1 to 7) categories of total age-adjusted ASQ-I score. Given that caregivers used their own definition of intelligence on the ladder scale, their ranking is a function of individual and community characteristics. Therefore, we constructed two sets of ASQ-I ranked percentiles: within communities and across the whole analytic sample.

#### Under- and over-estimation of child abilities

A difference score was calculated between the ranks of caregiver-perceived child intelligence and the ASQ-I. This score ranged from − 5 to 6 for both the within-community and whole samples. A positive score indicates that a caregiver ranked the child higher on the perceived intelligence scale than the child ranked on the ASQ-I. Given that small differences between the ranks may not be meaningful, we categorized these scores into under-estimation (difference less than − 2), matched (difference between − 1 and 1), and over-estimation (difference greater than 2).

### Covariates

#### Child characteristics

We examined mean-centered child age (in months), gender, and birth order with caregiver under- and over-estimation. We also included height-for-age z-score (HAZ) and weight-for-age z-score (WAZ), measured according to the World Health Organization (WHO) Child Growth reference standards [34]. The WHO defines stunting and underweight as having a HAZ and WAZ, respectively, two standard deviations below the median of same age and sex of a global reference population [34].

#### Caregiver characteristics

All caregiver variables are for the primary caregiver of the child. In the majority of cases, the primary caregiver was the mother, but in some, an older relative was the primary caregiver. We included mean-centered caregiver age (in years), education, depression, and belief of own influence on child intelligence in our analyses. We classified caregiver education into (1) Did not attend school; (2) Primary or less; (3) Secondary or Higher. Caregiver depression was measured using an adapted version of the Center for Epidemiologic Studies Depression Scale (CESD), a screening tool used to assess depressive symptoms [35]. The CESD was adapted, translated, and tested before use. The ten-item scale examines depressed affect, somatic symptoms, and positive affect. Items were summed and standardized to create a depression score. A higher score indicates that a caregiver has more depressive symptoms. Caregiver perceived influence on child intelligence was measured by asking “How much does your child’s intelligence depend on you?” and categorized into (1) None; (2) Some; (3) A lot.

#### Household characteristics

We examined the associations of household size, wealth, and stimulation and early learning opportunities with caregiver under- and over-estimation of child development. Household size was reported by the caregiver as the total number of individuals living in the household. To assess household wealth, an asset index was created using principal components analysis [36, 37]. Items in the principal components analysis included housing materials, electricity, toilet, drinking water source, personal property, and livestock. The first component was retained and quintiles of the wealth index were created. We assessed the stimulation and learning opportunities of a household using the Family Care Indicator (FCI) Scale, which has been previously used in a low-resource setting [38]. Using factor analysis, we created an FCI score based on stimulation activities, play materials, and books. Additionally, we included treatment arm and region indicators as control variables.

### Statistical analysis

To describe the agreement between caregiver-perceived child intelligence and assessed abilities, we used the Kendall *τ*_*b*_ correlation test, a non-parametric measure of the association between two ordinal variables that accounts for tied data [39].

We conducted multivariable multinomial logistic regressions to examine the child, caregiver, and household characteristics that were associated with caregiver under- and over-estimation of child abilities, setting matched as the reference outcome category. Model (1) included HAZ while Model (2) included WAZ. We used two different samples to analyze discordance: a within-community sample in which ASQ-I percentiles were ranked within communities and a whole sample in which percentiles were ranked across the whole analytic sample. A multinomial model was favored because the test for the proportional odds assumption was violated [40, 41]. Given the high correlation between HAZ and WAZ (Pearson’s correlation coefficient, r- = 0.71), we included them in separate models.

We examined collinearity between independent variables, not including HAZ and WAZ, using Pearson’s correlation coefficients and variance inflation factors. The highest correlation, using Pearson’s correlation coefficient, was between birth order and maternal age (*r* = 0.65). Multicollinearity was assessed using variance inflation factors (VIF), with a VIF greater than 10 indicative of severe collinearity between independent variables [42]. No independent variable had a VIF greater than three.

Analyses were repeated using only the interviewer-observed items of ASQ-I in order to examine the potential of reporting bias by participants. Ranked percentiles of these items were created within communities and multinomial logistic regressions were performed using this sample. Participants with missing data for any variable after imputation were excluded from analyses. Odds ratios (OR) and 95% confidence intervals (CI) were reported.

## Results

**Fig. 1:**
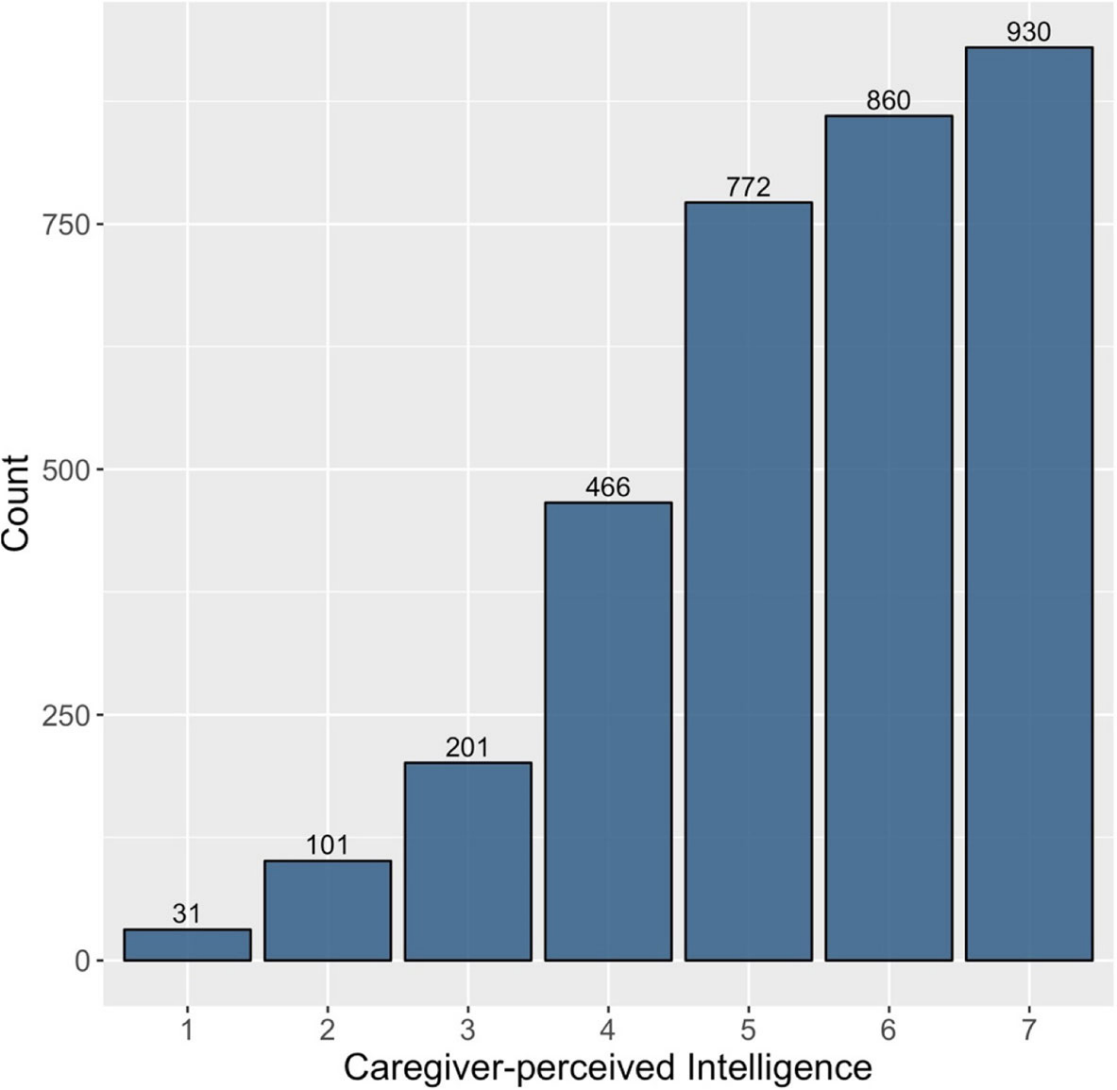
Caregiver-perceived Intelligence

The average age of children in our sample was 29 months; over 64% of children were stunted (Table 1). The average age of caregivers was 29 years and the majority (53%) of caregivers attended primary school or less. The distribution of perceived child intelligence was heavily left-skewed, with a large majority of caregivers perceiving their children to be more intelligent compared to other children in the community (Fig. 1).

**Table 1:** Cohort Characteristics, Madagascar, *N* = 3361

	Mean (SD; range)	% (N)
*Child Characteristics*		
Age (months)	29.38 (5.15; 16–42)	
Female		50.28 (1690)
Birth Order	2.64 (1.25; 1–4)	
HAZ	−2.34 (1.05; − 5.99-3.53)	
WAZ	−1.56 (0.93; − 4.94-2.45)	
Stunted (HAZ < − 2)		63.37 (2130)
Wasted (WHZ < − 2)		4.08 (137)
Underweight (WAZ < − 2)		30.17 (1014)
ASQ-I age-standardized score	0.00 (1.23; −4.93-4.16)	
ASQ-I ranked scores [Whole sample]		
≤ −1.42		14.31 (481)
-1.41 to −0.62		14.28 (480)
-0.61 to −0.10		14.04 (472)
-0.11 to 0.34		14.31 (481)
0.35 to 0.75		14.25 (479)
0.76 to 1.27		14.25 (479)
≥ 1.28		14.55 (489)
Perceived Child Intelligence Ladder		
1		0.92 (31)
2		3.01 (101)
3		5.98 (201)
4		13.86 (466)
5		22.97 (772)
6		25.59 (860)
7		27.67 (930)
*Caregiver Characteristics*		
Age (years)	28.57 (7.78; 15–83)	
Education		
Did not attend school		25.02 (841)
Primary or less		52.93 (1779)
Secondary or higher		22.05 (741)
CESD Depression score	17.15 (3.40; 10–29)	
Perceived influence on child’s intelligence		
None		14.67 (493)
Some		25.59 (860)
A lot		59.74 (2008)
*Household Characteristics*		
Household Size	6.52 (2.67; 2–21)	
Family Care Indicator Score	0.04 (0.84; −1.28-3.15)	
Wealth Quintiles		
Q1 (lowest)		20.17 (678)
Q2		19.99 (672)
Q3		19.70 (662)
Q4		19.82 (666)
Q5 (highest)		20.32 (683)

*Abbreviations*: *HAZ* Height-for-age z-score, *WHZ* Weight-for-height z-score, *WAZ* Weight-for-age z-score, *ASQ-I* Ages and Stages Questionnaire-Inventory, *CESD* Center for Epidemiologic Studies Depression Scale

**Fig. 2:**
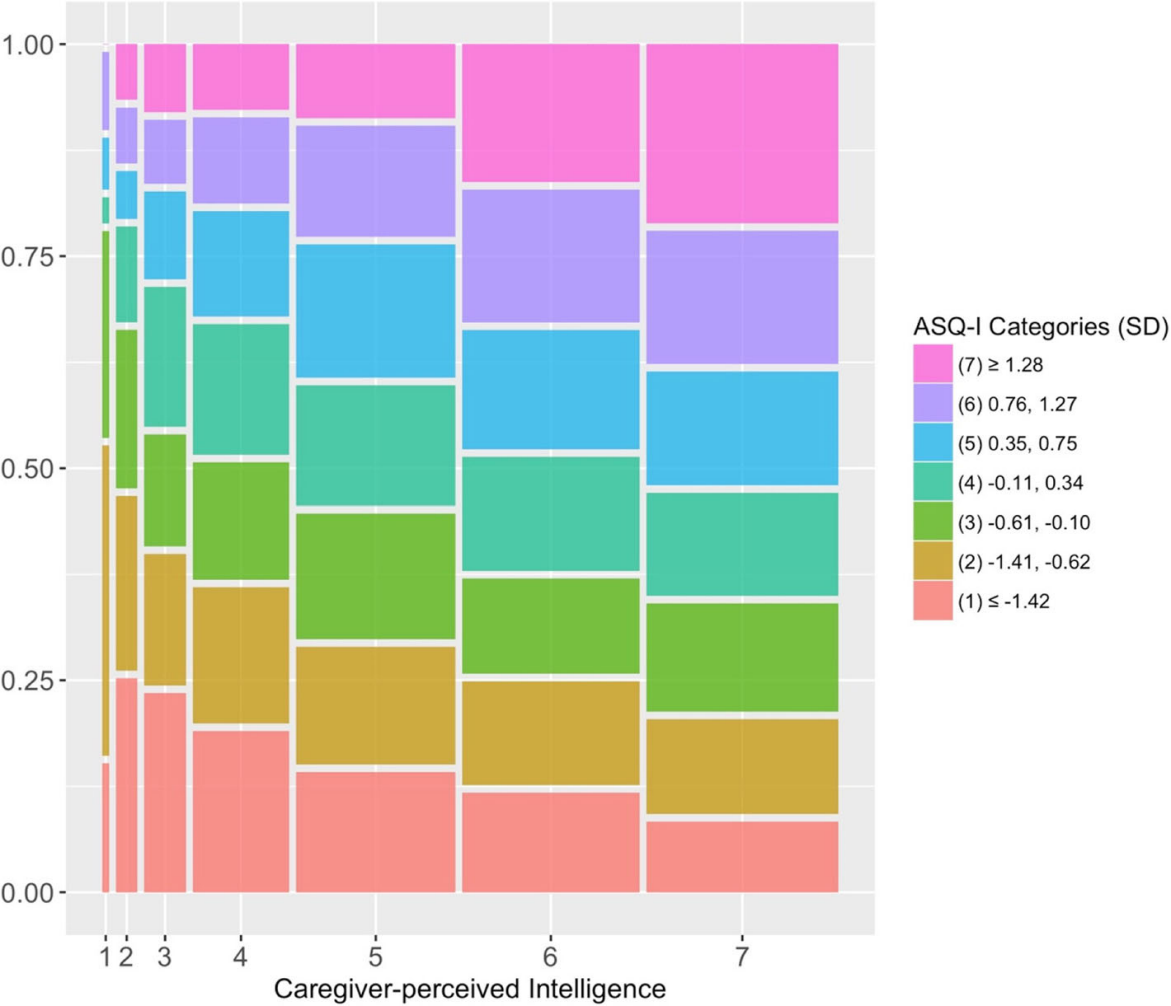
ASQ-I rankings vs. Caregiver-perceived Intelligence. ASQ-I categories were created using the age-standardized ASQ-I score and are presented in standard deviations (SD)

The correlation between caregiver-perceived child intelligence and ASQ-I score was weak (within-community sample: Kendall’s *τ*_*b*_ = 0.15, Asymptotic Standard Error = 0.013, *p* < 0.001; whole sample: *τ*_*b*_ = 0.17, Asymptotic Standard Error = 0.013, *p* < 0.001). A mosaic plot depicting the joint distribution of caregiver-perceived child intelligence and ASQ-I demonstrated that while a majority of caregivers ranked their child greater than five on the perceived intelligence scale, within each category of perceived intelligence, there was a wide range of child developmental abilities (Fig. 2). For example, among the 930 caregivers who ranked their children at the highest rung of the ladder (7), the ASQ-I scores for their children spanned the full range; however, of the children in the top rung of perceived intelligence, fewer than 32% had ASQ-I scores greater than 1 standard deviation above the mean. This demonstrated that in the highest perceived category, many caregivers over-estimated their children’s abilities.

We analyzed predictors of caregiver under- and over-estimation using two samples (within-community and whole) and present results by each sample.

### Within-community sample

Using ASQ-I ranked percentiles within communities, 8.2% (*N* = 275) of caregivers under-estimated and 49.3% (*N* = 1655) over-estimated their children’s abilities. In contrast, 42.6% (*N* = 1431) caregivers had matched scores.

#### Within-community under-estimation

Child age was associated with a small, decreased odds in under-estimation of child abilities (Table 2, HAZ model: OR = 0.97, 95% CI: 0.94–0.99; WAZ model: OR = 0.96, 95% CI: 0.94–0.99). Caregivers who believed they have a lot of influence on their child’s intelligence had lower odds of under-estimation compared to those who did not believe they have any influence, controlling for all other covariates (HAZ and WAZ models: OR = 0.68, 95% CI: 0.49–0.94).

**Table 2:** Correlates of caregiver under- and over-estimation using the within-community sample, Madagascar, 2016, *N* = 3361

	Model (1)		Model (2)	
	Under-estimation	Over-estimation	Under-estimation	Over-estimation
	OR (95% CI)	OR (95% CI)	OR (95% CI)	OR (95% CI)
*Child Characteristics*				
Age (months)	0.97** (0.94–0.99)	1.01 (0.99–1.02)	0.96** (0.94–0.99)	1.00 (0.99–1.02)
Gender	0.89 (0.70–1.12)	1.05 (0.92–1.19)	0.88 (0.69–1.11)	1.00 (0.88–1.14)
Birth Order	0.99 (0.85–1.14)	1.01 (0.94–1.08)	0.99 (0.85–1.14)	1.01 (0.94–1.08)
HAZ	0.93 (0.83–1.05)	0.71** (0.66–0.76)	–	–
WAZ	–	–	0.99 (0.87–1.13)	0.80** (0.74–0.85)
*Caregiver Characteristics*				
Age (years)	0.99 (0.96–1.01)	1.00 (0.99–1.01)	0.99 (0.96–1.01)	1.00 (0.99–1.01)
Education				
No school [ref]	1.00	1.00	1.00	1.00
Primary or less	1.07 (0.76–1.51)	0.95 (0.77–1.18)	1.07 (0.76–1.51)	0.96 (0.78–1.19)
Secondary or Higher	0.92 (0.57–1.48)	0.70** (0.54–0.91)	0.92 (0.57–1.49)	0.72* (0.56–0.93)
Depression Score	1.01 (0.86–1.19)	1.01 (0.93–1.10)	1.01 (0.86–1.19)	1.02 (0.94–1.10)
Belief of influence on child intelligence				
None [ref]	1.00	1.00	1.00	1.00
Some	0.94 (0.66–1.35)	0.97 (0.77–1.21)	0.95 (0.66–1.36)	0.97 (0.78–1.22)
A lot	0.68* (0.49–0.94)	0.99 (0.80–1.22)	0.68* (0.49–0.94)	0.99 (0.81–1.22)
*Household Characteristics*				
Household Size	1.04 (0.98–1.10)	1.01 (0.98–1.04)	1.04 (0.98–1.10)	1.02 (0.99–1.05)
Family Care Indicator Score	0.96 (0.80–1.16)	1.00 (0.89–1.12)	0.96 (0.79–1.15)	0.98 (0.88–1.10)
Wealth Quintiles				
Q1 (lowest) [ref]	1.00	1.00	1.00	1.00
Q2	0.80 (0.51–1.25)	0.91 (0.71–1.15)	0.80 (0.51–1.25)	0.92 (0.73–1.17)
Q3	0.84 (0.54–1.32)	1.06 (0.84–1.33)	0.84 (0.54–1.31)	1.06 (0.84–1.35)
Q4	0.95 (0.59–1.52)	0.90 (0.71–1.13)	0.94 (0.59–1.50)	0.91 (0.73–1.14)
Q5 (highest)	0.71 (0.43–1.17)	0.95 (0.73–1.23)	0.70 (0.42–1.16)	0.95 (0.73–1.24)

Multinomial logistic regression with matched caregiver-perceived child intelligence and ASQ-I ranking as the reference outcome category. All estimations adjusted for treatment arm and region, and corrected for clustering at the village level. Model (1) included height-for-age z-score while Model (2) included weight-for-age z-score.

** *p* < 0.01, * *p* < 0.05

#### Within-community over-estimation

For every standard deviation increase in HAZ, the odds of caregiver over-estimation decreased 29% (OR = 0.71, 95% CI: 0.66-0.76), controlling for child, caregiver, and household covariates. Similarly, for every standard deviation increase in WAZ, the odds of caregiver over-estimation decreased 20%, after controlling for all covariates (OR = 0.80, 95% CI: 0.74–0.85). In model (1) with HAZ, caregivers with secondary or higher education had 0.70 times the odds of over-estimation compared to those who did not attend school (95% CI: 0.54–0.91). Similar results were found in model (2) with WAZ (OR = 0.72, 95% CI: 0.56–0.93).

### Whole sample

When we examined ASQ-I percentiles across the whole sample, we found that 7.8% (*N* = 260) of caregivers under-estimated and 47.1% (*N* = 1583) over-estimated their child’s abilities, while 45.2% (*N* = 1518) of caregivers had matched scores.

#### Whole sample under-estimation

Child age remained significantly associated with lower odds of under-estimation, after controlling for all covariates (Table 3, HAZ and WAZ models: OR = 0.95, 95% CI: 0.92–0.98). Households in the highest wealth quintile had decreased odds of under-estimation compared to the lowest quintile (HAZ model: OR = 0.53, 95% CI: 0.31–0.88; WAZ model: OR = 0.51, 95% CI: 0.31–0.86).

**Table 3:** Correlates of caregiver under- and over-estimation using the whole sample, Madagascar, 2016, *N* = 3361

	Model (1)		Model (2)	
	Under-estimation	Over-estimation	Under-estimation	Over-estimation
	OR (95% CI)	OR (95% CI)	OR (95% CI)	OR (95% CI)
*Child Characteristics*				
Age (months)	0.95** (0.92–0.98)	1.01 (1.00–1.03)	0.95** (0.92–0.98)	1.01 (0.99–1.03)
Gender	0.87 (0.67–1.11)	1.00 (0.88–1.13)	0.87 (0.67–1.12)	0.98 (0.86–1.11)
Birth Order	0.99 (0.86–1.14)	1.01 (0.94–1.09)	0.99 (0.86–1.14)	1.01 (0.94–1.09)
HAZ	1.09 (0.96–1.25)	0.82** (0.75–0.88)	–	–
WAZ	–	–	1.13 (0.98–1.31)	0.85** (0.79–0.92)
*Caregiver Characteristics*				
Age (years)	0.99 (0.97–1.02)	1.00 (0.99–1.02)	0.99 (0.97–1.02)	1.00 (0.99–1.01)
Education				
No school [ref]	1.00	1.00	1.00	1.00
Primary or less	1.04 (0.73–1.48)	0.80* (0.65–0.98)	1.03 (0.72–1.47)	0.80* (0.65–0.99)
Secondary or Higher	1.15 (0.72–1.84)	0.64** (0.49–0.84)	1.13 (0.70–1.82)	0.65** (0.49–0.85)
Depression Score	1.12 (0.95–1.31)	1.05 (0.96–1.15)	1.11 (0.95–1.31)	1.05 (0.96–1.15)
Belief of influence on child intelligence				
None [ref]	1.00	1.00	1.00	1.00
Some	0.72 (0.49–1.06)	0.81 (0.63–1.05)	0.72 (0.49–1.06)	0.81 (0.63–1.06)
A lot	0.71 (0.49–1.03)	0.72** (0.58–0.91)	0.70 (0.49–1.02)	0.73** (0.58–0.91)
*Household Characteristics*				
Household Size	1.01 (0.95–1.07)	1.00 (0.97–1.03)	1.01 (0.95–1.07)	1.00 (0.97–1.03)
Family Care Indicator Score	0.96 (0.77–1.20)	1.04 (0.88–1.23)	0.97 (0.78–1.20)	1.03 (0.88–1.22)
Wealth Quintiles				
Q1 (lowest) [ref]	1.00	1.00	1.00	1.00
Q2	0.88 (0.60–1.31)	1.16 (0.88–1.52)	0.87 (0.59–1.29)	1.18 (0.90–1.54)
Q3	0.80 (0.53–1.21)	1.17 (0.87–1.58)	0.80 (0.53–1.20)	1.18 (0.87–1.59)
Q4	0.72 (0.47–1.11)	0.86 (0.65–1.15)	0.71 (0.46–1.10)	0.87 (0.66–1.16)
Q5 (highest)	0.53* (0.31–0.88)	0.73 (0.53–1.02)	0.51* (0.31–0.86)	0.74 (0.53–1.03)

Multinomial logistic regression with matched caregiver-perceived child intelligence and ASQ-I ranking as the reference outcome category. All estimations adjusted for treatment arm and region, and corrected for clustering at the village level. Model (1) included height-for-age z-score while Model (2) included weight-for-age z-score.

** *p* < 0.01, * *p* < 0.05

#### Whole sample over-estimation

Similar to findings in the within-community sample analyses, for every standard deviation increase in HAZ, the odds of over-estimation decreased 18% (Table 3, OR = 0.82, 95% CI: 0.75–0.88), and for every standard deviation increase in WAZ, the odds of over-estimation decreased 15%, after adjusting for all other covariates (OR = 0.85, 95% CI: 0.79–0.92). Additionally, in the model with HAZ, caregivers with primary school or less had 0.80 times the odds of over-estimation compared to those who did not attend school (95% CI: 0.65–0.98). This association further decreased when comparing caregivers with secondary education or higher and those who did not attend school, adjusting for all other covariates (OR = 0.64, 95% CI: 0.49–0.84). Similar results and trends were found in the WAZ models (primary school or less vs. no school: OR = 0.80, 95% CI: 0.65–0.99; secondary school or higher vs. no school: OR = 0.65, 95% CI: 0.49–0.85). Caregivers who believed they had a lot of influence on their child’s development had lower odds of over-estimation than those who did not believe they have any influence, after adjusting for all covariates (HAZ model: OR = 0.72, 95% CI: 0.58–0.91; WAZ model: OR = 0.73, 95% CI: 0.58–0.91).

### Sensitivity analyses

When we limited ASQ-I score to the interviewer-observed items and created ranked percentiles within communities, 8.4% of caregivers under-estimated (*N* = 282) and 49.8% over-estimated (*N* = 1675), while 41.8% (*N* = 1404) had matched scores. In adjusted multinomial models using this sample, we found similar results to the within-community sample analysis (Additional file 1). Greater child age and greater caregiver belief of influence on child intelligence remained associated with lower odds of under-estimation in both HAZ and WAZ models. Greater caregiver education, HAZ, and WAZ were each associated with decreased odds of over-estimation. The only difference in findings compared to the within-community sample was that caregivers in the highest wealth quintile had lower odds of under-estimation compared to the lowest quintile, adjusting for all other child, caregiver, and household level covariates (HAZ model: OR = 0.63, 95% CI: 0.41–0.99; WAZ model: OR = 0.61, 95% CI: 0.39–0.96).

## Discussion

In this study in rural Madagascar, caregiver perceptions of child intelligence were not consistent with the ASQ-I, an assessment of child development. More than half (57.5%) of Malagasy women ranked their children’s intelligence differently than how their children were ranked based on the ASQ-I. Specifically, about 8.2% of caregivers under-estimated and 49.3% over-estimated their children’s development relative to others. We found similar estimates when we used ASQ-I percentiles across the whole sample (7.8 and 47.1%, respectively) and in our sensitivity analysis when we restricted to using the interviewer-observed items of the ASQ-I (8.4 and 49.8%, respectively). Our findings are consistent with the limited published literature on parental perceptions of child nutrition in that there is often a discordance between caregiver perceptions and more objective measures [19–23]. The inconsistency between perceived child intelligence and the assessment of child developmental abilities may be unsurprising given that caregivers may use different indicators to rank their child in terms of intelligence compared to the ASQ-I indicators typically used to assess developmental abilities. For example, a qualitative study in Malawi on perceptions of child development found that in addition to motor and language milestones, social milestones related to completing chores and taking on community leadership roles were also salient for communities [43], but are not included in the ASQ-I. Even so, our findings highlight a need for further inquiry into how caregivers conceptualize child abilities at a young age and what, if any, impacts there may be of this discordance on uptake of behavior change interventions.

Parents with children with better nutritional status, measured using height-for-age z-score and weight-for-age z-score, were more accurate at estimating their child’s abilities. This finding was consistent across all three samples (within-community, whole, and ASQ-I interviewer-observed items only), after controlling for key child-, caregiver-, and household-level covariates. Our findings suggest that caregivers who use height or weight as a visual cue to benchmark their child’s development compared to other children may be more accurate. Research from Guatemala documented how parents’ perceptions about what constitutes ‘normal’ height at age two influenced feeding practices and may be a function of the height distribution of children living around them in the locality [18]. Given that stunting has been associated with poorer child cognitive development, it may be that in this population, where over 60% of children are stunted, caregivers may be using relative height among children as an indicator of child health and development. The same may also be true for caregivers who use relative weight among children in their communities. Future research is needed to examine whether parental perceptions of child growth predicts child development. Nevertheless, given the strong link between optimal infant and young child feeding practices and early child development, our results suggest the potential benefit of integrating nutrition counseling and child stimulation messages in parenting programs that aim to improve child development. Many existing interventions have accomplished this by integrating responsive feeding with talking with infants and connecting the importance between healthy, nutritious food and child growth and intelligence. However, parents who have inaccurate perceptions of their child’s development may be less likely to incorporate such behavior changes in their daily lives if they believe their child is on an adequate developmental trajectory. Therefore, there may be added benefit for child development programs to incorporate direct feedback to parents on their child’s trajectory as suggested by work in Malawi [17]. This finding may assist in delivering additional support to those who may be misperceiving their child’s development and in assessing program implementation success.

Caregivers with a greater belief that they have an influence on child intelligence were less likely to under-estimate in the within-community and ASQ-I interviewer-observed only samples. In the whole sample, caregivers with a greater belief of their influence were more accurate in estimating their child’s abilities. In either case, caregivers with a stronger belief that they can affect their child’s intelligence perceived their child’s intelligence in comparable ways to children’s estimated abilities. If we consider answers to the question “How much does your child’s intelligence depend on you?” as a proxy for caregiver self-efficacy, those who believe they have more agency are better at identifying their child’s abilities. We may also consider the question as a proxy for caregiver belief that intelligence is flexible and growing. Parents who have greater self-efficacy or believe intelligence is malleable may be more likely to engage in positive parenting practices, which can lead to improved child cognitive and socioemotional development, and also points to the potential to modify parental self-efficacy through interventions [44, 45]. In addition, knowledge of child development is a key component to whether parental self-efficacy affects parenting practices. For instance, among mothers with premature infants in Baltimore, Maryland, parental self-efficacy and parenting competence was positively associated with ECD measures only when there was high parental knowledge of infant development [46]. Given the growing focus on child development in low-resource contexts, future research to understand caregiver self-efficacy, beliefs of child intelligence, and parenting knowledge could identify potential barriers that inhibit behavior change related to child stimulation and development and potential solutions to motivate caregivers.

Additionally, we found that the discordance between caregiver-perceived child intelligence and ASQ-I was lower among caregivers of higher socioeconomic status. Those with secondary or higher education were less likely to over-estimate their child’s development and those with greater wealth were less likely to under-estimate. This finding is consistent with the documented socioeconomic gradients and child development in Madagascar such that even in the context of extreme poverty, children from families with greater wealth and higher education performed better on a set of child development measures [47]. Our findings suggest that socioeconomic status may affect caregiver beliefs of child abilities when ranked with respect to other children. In Malawi, poorer and less educated parents had less accurate beliefs about their children’s academic performance; however, after they were provided with clear information about their child’s performance in school, less educated parents updated their beliefs more than higher educated parents and changed their educational investments [17]. Barriers to accessing school performance information may contribute to inaccurate perceptions among poorer households. In order to mitigate differential educational decisions by socioeconomic status, it is likely important to understand parental perceptions of their child’s abilities before they start school. Future research should examine how caregiver perceptions of child intelligence and development vary across socioeconomic status and how these differential beliefs may affect investments in education and home environment in order to target behavior change messaging to caregivers with inaccurate perceptions and encourage educational investments for children early in life to prepare them for school.

Some limitations of the current study warrant discussion. First, this study used cross-sectional data to examine determinants of the discordance between caregiver-perceived child intelligence and child developmental abilities assessed with the ASQ-I; therefore, we do not claim causality. Second, we captured caregiver-perceived child intelligence through one rank-based question with an open interpretation of intelligence. We acknowledge that caregiver perceptions of child intelligence and assessments of children’s developmental abilities are context-dependent. Indeed, Harkness et al. emphasize the important link between culture and early child development, noting that settings, customs, and caretaker ethnotheories, or parents’ culturally constructed beliefs about child behaviors and development, operate together in a system that continually change and ultimately influence early child development [48]. Future work should incorporate qualitative methods to identify beliefs of child development [43] and consider applying vignettes to anchor the perceived ladder scale [49]. Third, we were unable to assess early child development through direct observation due to cost and time constraints and we acknowledge the potential for recall and social desirability bias in caregiver report; however, when we limited our analysis to the interviewer-observed items of the ASQ-I, our results did not substantially change.

Our study quantitatively compared caregiver perceptions of child intelligence with an assessment of child development in a low-income country using a perceived ranking scale and examined correlates of discordance. Previous quantitative studies have used the Neonatal Perception Inventory to measure maternal perceptions of early child development [10, 11]. This measure assesses parents’ perceptions of the newborn relative to their concept of an average newborn in relation to crying, vomiting, sleeping, feeding, and predictability [50, 51]. Others have used the Knowledge of Infant Development Inventory, which examines parents’ knowledge of child-rearing practices, developmental processes, developmental milestones, and health and safety concerns [46, 52]. However, these measures primarily focus on early infancy, but early child development extends well beyond this stage. While previous studies have identified that parental perceptions of child health diverge from objective measures, we examined a wide range of child, caregiver, and household factors on caregiver estimation of child developmental abilities. Additionally, our results are robust to using interviewer-observed items of the ASQ-I in a sensitivity analysis, suggesting that reporting bias was not a major issue in our analysis.

## Conclusion

Our findings demonstrate a discordance between caregiver perceptions of child intelligence and an early child development measure in a low-income, rural Malagasy population. With the increasing number of parenting interventions and programs in low-income contexts, it is important to consider caregiver perceptions of children’s developmental abilities early in life and how these perceptions affect educational investments. Future work should examine whether providing specific feedback on child developmental milestones to parents would alter caregiving or investment practices. Additionally, understanding what factors influence perceptions can provide information on common cues caregivers use on a daily basis to assess child development, which can inform behavior change interventions. Further investigation is needed to characterize how these may differ with researcher-observed measures in low-income contexts.

## Additional file

Additional file 1: Correlates of caregiver under- and over-estimation using the interviewer-observed items only, Madagascar, 2016, *N* = 3361. (DOCX 16 kb)

## Data Availability

The data used during the current study are available from the corresponding author on reasonable request.

## Abbreviations

ASQ: Ages and Stages Questionnaire
ASQ-I: Ages and Stages Questionnaire: Inventory
CESD: Center for Epidemiologic Studies Depression Scale
CI: Confidence interval
ECD: Early child development
HAZ: Height-for-age z-score
OR: Odds ratio
VIF: Variance inflation factor
WAZ: Weight-for-age z-score
WHO: World Health Organization
WHZ: Weight-for-height z-score

## References

[1] Black MM, Walker SP, Fernald LCH, Andersen CT, DiGirolamo AM, Lu C, McCoy DC, Fink G, Shawar YR, Shiffman J (2017). Early childhood development coming of age: science through the life course. Lancet.

[2] Grantham-McGregor S, Cheung YB, Cueto S, Glewwe P, Richter L, Strupp B (2007). International child development steering G: developmental potential in the first 5 years for children in developing countries. Lancet.

[3] Walker SP, Wachs TD, Grantham-McGregor S, Black MM, Nelson CA, Huffman SL, Baker-Henningham H, Chang SM, Hamadani JD, Lozoff B (2011). Inequality in early childhood: risk and protective factors for early child development. Lancet.

[4] Luby JL (2015). Poverty’s most insidious damage: the developing brain. JAMA Pediatr.

[5] Shonkoff JP, Garner AS (2012). Committee on psychosocial aspects of C, family H, committee on early childhood a, dependent C, section on D, behavioral P: the lifelong effects of early childhood adversity and toxic stress. Pediatrics.

[6] Bornstein MH, Bradley RH (2014). Socioeconomic status, parenting, and child development: Routledge.

[7] Huang K-Y, Caughy MOB, Genevro JL, Miller TL (2005). Maternal knowledge of child development and quality of parenting among white, African-American and Hispanic mothers. J Appl Dev Psychol.

[8] Grusec JE (2011). Socialization processes in the family: social and emotional development. Annu Rev Psychol.

[9] Benasich AA, Brooks-Gunn J (1996). Maternal attitudes and knowledge of child-rearing: associations with family and child outcomes. Child Dev.

[10] Scher A, Tirosh E (1997). Early maternal perceptions and child development: a comparison between two subgroups in Israel. J Reprod Infant Psychol.

[11] Hernández-Martínez C, Canals Sans J, Fernández-Ballart J (2011). Parents' perceptions of their neonates and their relation to infant development. Child Care Health Dev.

[12] Olson SL, Bates JE, Bayles K (1989). Predicting Long-term developmental outcomes from maternal perceptions of infant and toddler behavior. Infant Behav Dev.

[13] Pauli-Pott U, Mertesacker B, Bade U, Haverkock A, Beckmann D (2003). Parental perceptions and infant temperament development. Infant Behav Dev.

[14] Olson SL, Bates JE, Sandy JM, Lanthier R (2000). Early developmental precursors of externalizing behavior in middle childhood and adolescence. J Abnorm Child Psychol.

[15] Kinsler J, Pavan R (2016). Parental beliefs and investment in children: The distortionary impact of schools.

[16] Ogunnaike OA, Houser RF (2002). Yoruba toddlers' engagement in errands and cognitive performance on the Yoruba mental subscale. Int J Behav Dev.

[17] Dizon-Ross R (2018). Parents’ Beliefs About Their Children's Academic Ability: Implications for Educational Investments. National Bureau of Economic Research.

[18] Wang F, Puentes E, Behrman J, Cunha F (2018). You are what your parents think: height and local reference points.

[19] Bertrand WE, Walmus BF (1983). Maternal knowledge, attitudes and practice as predictors of diarrhoeal disease in young children. Int J Epidemiol.

[20] Hochdorn A, Baldi I, Paramesh EC, Kumar M, Gulati A, Gregori D (2014). Is my kid out of size? Indian mothers’ desirability Bias in evaluation of their Children’s weight. Indian J Pediatr.

[21] Mwangome MK, Fegan G, Prentice AM, Berkley JA (2015). Maternal perception of malnutrition among infants using verbal and pictorial methods in Kenya. Public Health Nutr.

[22] Roy SK, Rahman MM, Mitra AK, Ali M, Alam AN, Akbar MS (1993). Can mothers identify malnutrition in their children?. Health Policy Plan.

[23] Syahrul S, Kimura R, Tsuda A, Susanto T, Saito R, Agrina A (2017). Parental perception of the Children’s weight status in Indonesia. Nurs Midwifery Stud.

[24] World Bank (2017). World development indicators.

[25] WHO: World Health Statistics 2017 (2017). Monitoring health for the SDGs, Sustainable Development Goals.

[26] Thompson RA, Nelson CA (2001). Developmental science and the media. Early brain development. Am Psychol.

[27] Fernald LCH, Galasso E, Qamruddin J, Ranaivoson C, Ratsifandrihamanana L, Stewart CP, Weber AM (2016). A cluster-randomized, controlled trial of nutritional supplementation and promotion of responsive parenting in Madagascar: the MAHAY study design and rationale. BMC Public Health.

[28] Adler NE, Epel ES, Castellazzo G, Ickovics JR (2000). Relationship of subjective and objective social status with psychological and physiological functioning: preliminary data in healthy white women. Health Psychol.

[29] Singh-Manoux A, Marmot MG, Adler NE (2005). Does subjective social status predict health and change in health status better than objective status?. Psychosom Med.

[30] Goodman E, Huang B, Schafer-Kalkhoff T, Adler NE (2007). Perceived socioeconomic status: a new type of identity that influences adolescents’ self-rated health. J Adolesc Health.

[31] Ritterman Weintraub ML, Fernald LC, Adler N, Bertozzi S, Syme SL (2015). Perceptions of social mobility: development of a new psychosocial indicator associated with adolescent risk behaviors. Front Public Health.

[32] Clifford J, Chen C-I, Xie H, Chen C-Y, Murphy K, Ascetta K, Frantz R, Hansen S (2018). Examining the technical adequacy of the ages & stages questionnaires: INVENTORY. Infants Young Child.

[33] Xie H, Clifford J, Squires J, Chen CY, Bian X, Yu Q (2017). Adapting and validating a developmental assessment for chinese infants and toddlers: the ages & stages questionnaires: Inventory. Infant Behav Dev.

[34] WHO Multicentre Growth Reference Study Group (2006). WHO child growth standards based on length/height, weight and age. Acta Paediatr Suppl.

[35] Radloff LS (1977). The CES-D scale: a self-report depression scale for research in the general population. Appl Psychol Meas.

[36] Filmer D, Pritchett LH (2001). Estimating wealth effects without expenditure data--or tears: an application to educational enrollments in states of India. Demography.

[37] Vyas S, Kumaranayake L (2006). Constructing socio-economic status indices: how to use principal components analysis. Health Policy Plan.

[38] Hamadani JD, Tofail F, Hilaly A, Huda SN, Engle P, Grantham-McGregor SM (2010). Use of family care indicators and their relationship with child development in Bangladesh. J Health Popul Nutr.

[39] Agresti A (2010). Analysis of ordinal categorical data.

[40] Brant R (1990). Assessing proportionality in the proportional odds model for ordinal logistic regression. Biometrics.

[41] Long JS, Freese J (2006). Regression models for categorical dependent variables using Stata: Stata press.

[42] Fox J, Fox J (2016). Applied regression analysis and generalized linear models.

[43] Gladstone M, Lancaster G, Umar E, Nyirenda M, Kayira E, Van Den Broek N, Smyth RL (2010). Perspectives of normal child development in rural Malawi – a qualitative analysis to create a more culturally appropriate developmental assessment tool. Child Care Health Dev.

[44] Jones TL, Prinz RJ (2005). Potential roles of parental self-efficacy in parent and child adjustment: a review. Clin Psychol Rev.

[45] Haimovitz K, Dweck CS (2016). Parents’ views of failure predict Children’s fixed and growth intelligence mind-sets. Psychol Sci.

[46] Hess CR, Teti DM, Hussey-Gardner B (2004). Self-efficacy and parenting of high-risk infants: the moderating role of parent knowledge of infant development. J Appl Dev Psychol.

[47] Fernald LCH, Weber A, Galasso E, Ratsifandrihamanana L (2011). Socioeconomic gradients and child development in a very low income population: evidence from Madagascar. Dev Sci.

[48] Harkness S, Super C, Mavridis C, Barry O, Zeitlin M (2013). Culture and early childhood development: implications for policy and programs.

[49] Ravallion M, Himelein K, Beegle K (2016). Can subjective questions on economic welfare be trusted?. Econ Dev Cult Chang.

[50] Broussard ER, Hartner MS (1970). Maternal perception of the neonate as related to development. Child Psychiatry Hum Dev.

[51] Broussard ER (1979). Assessment of the adaptive potential of the mother-infant system: the neonatal perception inventories. Semin Perinatol.

[52] Bornstein MH, Cote LR, Haynes OM, Hahn C-S, Park Y (2010). Parenting knowledge: experiential and sociodemographic factors in European American mothers of young children. Dev Psychol.

