# Supplementary material for: Caregiver perceptions of child development in rural Madagascar: a cross-sectional study
Source: BMC Public Health. 2019 Sep 11;19:1256. doi: 10.1186/s12889-019-7578-3 (PMC6739938; doi:10.1186/s12889-019-7578-3)
Supplement: Supplementary file 1 — Correlates of caregiver under- and over-estimation using the interviewer-observed items only, Madagascar, 2016, N = 3361. (DOCX 16 kb) [file 12889_2019_7578_MOESM1_ESM.docx]

| Additional File 1. Correlates of caregiver under- and over-estimation using the interviewer-observed items only, Madagascar, 2016, N=3,361 | | | | | |
| --- | --- | --- | --- | --- | --- |
|  | Caregiver vs. Interviewer Ranking  Model (1) | | Caregiver vs. Interviewer Ranking  Model (2) | |  |
|  | Under-estimation | Over-estimation | Under-estimation | Over-estimation |  |
|  | OR (95% CI) | OR (95% CI) | OR (95% CI) | OR (95% CI) |  |
| *Child Characteristics* |  |  |  |  |  |
| Age (months) | 0.95** (0.93 - 0.98) | 1.00 (0.98 - 1.01) | 0.95** (0.93 - 0.98) | 0.99 (0.98 - 1.01) |  |
| Gender | 0.81 (0.64 - 1.03) | 1.12 (0.98 - 1.27) | 0.8 (0.63 - 1.02) | 1.09 (0.96 - 1.24) |  |
| Birth Order | 0.96 (0.85 - 1.09) | 1.03 (0.95 - 1.11) | 0.96 (0.85 - 1.09) | 1.03 (0.95 - 1.11) |  |
| Height-for-age z-score | 1.03 (0.91 - 1.16) | 0.79** (0.73 - 0.84) | -- | -- |  |
| Weight-for-age z-score | -- | -- | 1.12 (0.98 - 1.27) | 0.83** (0.76 - 0.90) |  |
| *Caregiver Characteristics* |  |  |  |  |  |
| Age (years) | 1.00 (0.98 - 1.02) | 1.01 (1.00 - 1.02) | 1.00 (0.98 - 1.02) | 1.01 (1.00 - 1.02) |  |
| Education |  |  |  |  |  |
| No school [ref] | 1.00 | 1.00 | 1.00 | 1.00 |  |
| Primary or less | 1.31 (0.94 - 1.83) | 1.00 (0.82 - 1.21) | 1.30 (0.93 - 1.82) | 1.00 (0.82 - 1.23) |  |
| Secondary or Higher | 1.03 (0.64 - 1.66) | 0.68** (0.53 - 0.86) | 1.01 (0.62 - 1.64) | 0.69** (0.54 - 0.88) |  |
| Depression Score | 1.00 (0.84 - 1.19) | 0.99 (0.91 - 1.08) | 1.00 (0.84 - 1.19) | 0.99 (0.91 - 1.08) |  |
| Belief of influence on child intelligence |  |  |  |  |  |
| None [ref] | 1.00 | 1.00 | 1.00 | 1.00 |  |
| Some | 0.81 (0.54 - 1.21) | 0.98 (0.78 - 1.23) | 0.81 (0.54 - 1.20) | 0.98 (0.78 - 1.24) |  |
| A lot | 0.63* (0.44 - 0.91) | 1.07 (0.86 - 1.32) | 0.63* (0.44 - 0.90) | 1.07 (0.86 - 1.33) |  |
| *Household Characteristics* |  |  |  |  |  |
| Household Size | 0.99 (0.93 - 1.05) | 0.98 (0.96 - 1.01) | 0.99 (0.93 - 1.05) | 0.99 (0.96 - 1.01) |  |
| Family Care Indicator Score | 1.04 (0.84 - 1.28) | 1.06 (0.95 - 1.18) | 1.03 (0.84 - 1.27) | 1.05 (0.94 - 1.16) |  |
| Wealth Quintiles |  |  |  |  |  |
| Q1 (lowest) [ref] | 1.00 | 1.00 | 1.00 | 1.00 |  |
| Q2 | 0.95 (0.65 - 1.40) | 1.01 (0.81 - 1.27) | 0.94 (0.65 - 1.38) | 1.03 (0.82 - 1.29) |  |
| Q3 | 0.75 (0.50 - 1.13) | 1.01 (0.80 - 1.28) | 0.75 (0.50 - 1.12) | 1.02 (0.81 - 1.29) |  |
| Q4 | 0.70 (0.46 - 1.06) | 0.93 (0.73 - 1.17) | 0.69 (0.45 - 1.05) | 0.94 (0.75 - 1.19) |  |
| Q5 (highest) | 0.63* (0.41 - 0.99) | 0.97 (0.76 - 1.24) | 0.61* (0.39 - 0.96) | 0.98 (0.77 - 1.26) |  |
| Multinomial logistic regression was performed with matched interviewer-based and caregiver-perceived ECD ranking as the reference outcome category. All estimations adjusted for treatment arm and region, and corrected for clustering at the village level. Model (1) included height-for-age z-score while Model (2) included weight-for-age z-score.  ** p<0.01, * p<0.05 | | | | | |
